# Deformability of Stored Red Blood Cells

**DOI:** 10.3389/fphys.2021.722896

**Published:** 2021-09-22

**Authors:** Gregory Barshtein, Ivana Pajic-Lijakovic, Alexander Gural

**Affiliations:** ^1^Biochemistry Department, The Faculty of Medicine, The Hebrew University of Jerusalem, Jerusalem, Israel; ^2^Department of Chemical Engineering, University of Belgrade, Belgrade, Serbia; ^3^Department of Hematology, Hadassah Hebrew University Medical Center, Jerusalem, Israel

**Keywords:** red blood cells, RBC deformability, transfusion, RBC storage lesion, RBC storage

## Abstract

Red blood cells (RBCs) deformability refers to the cells’ ability to adapt their shape to the dynamically changing flow conditions so as to minimize their resistance to flow. The high red cell deformability enables it to pass through small blood vessels and significantly determines erythrocyte survival. Under normal physiological states, the RBCs are attuned to allow for adequate blood flow. However, rigid erythrocytes can disrupt the perfusion of peripheral tissues and directly block microvessels. Therefore, RBC deformability has been recognized as a sensitive indicator of RBC functionality. The loss of deformability, which a change in the cell shape can cause, modification of cell membrane or a shift in cytosol composition, can occur due to various pathological conditions or as a part of normal RBC aging (*in vitro* or *in vivo*). However, despite extensive research, we still do not fully understand the processes leading to increased cell rigidity under cold storage conditions in a blood bank (*in vitro* aging), In the present review, we discuss publications that examined the effect of RBCs’ cold storage on their deformability and the biological mechanisms governing this change. We first discuss the change in the deformability of cells during their cold storage. After that, we consider storage-related alterations in RBCs features, which can lead to impaired cell deformation. Finally, we attempt to trace a causal relationship between the observed phenomena and offer recommendations for improving the functionality of stored cells.

## Introduction

The primary physiological role of red blood cells (RBCs) is the supply of oxygen to tissues. To accomplish this task, RBCs have unique flow-affecting properties, which play a crucial role in blood circulation in health and disease (Yedgar et al., 2002; Barshtein et al., 2007), and thereby define the RBC hemodynamic functionality, namely their capacity to affect blood circulation (Barshtein et al., 2018b). One of the main characteristics that determine the behavior of cells in the bloodstream is their deformability. RBC deformability is the cells’ ability to adapt their shape to the dynamically changing flow conditions to minimize their resistance to flow. This is particularly important for their passage through the capillaries, which are narrower than the RBCs. Reduced deformability (increased rigidity) results in impaired perfusion and oxygen delivery to peripheral tissues (Parthasarathi and Lipowsky, 1999; Sakr et al., 2007; Matot et al., 2013), because rigid RBCs can directly block capillaries (McHedlishvili, 1998). RBC deformability is also a significant determinant of their ability to pass through the splenic vasculature; thus, reduced deformability, which is always the case in aged RBCs, hinders their transit and increases splenic RBC sequestration and destruction (Warkentin et al., 1990; Mohandas and Chasis, 1993; An and Mohandas, 2008; Huang et al., 2014).

Under normal physiological conditions, the RBC deformability enables adequate blood flow. However, under cold storage conditions (*in vitro* aging), cells lose their deformability, and this alteration can influence their after-transfusion performance (Matot et al., 2013; Barshtein et al., 2016, 2017). The observed increase in the stiffness of RBCs is usually associated with changes undergone by the cell membrane and the cytosol; in particular, alteration of their shape, elevation of the membrane rigidity, and the cytosol viscosity (Dao et al., 2003; Kuzman et al., 2004; Huisjes et al., 2018).

This review analyzes the effect of RBCs’ cold storage on their deformability and the biological mechanisms governing this change. We first discuss the change in the deformability of cells during their cold storage. After that, we consider storage-related alterations in RBCs features, which can lead to impaired cell deformation. Finally, we attempted to trace a causal relationship between the observed phenomena and offered recommendations for improving the functionality of stored cells.

## Cold Storage of RBCs and Cell Deformability

It was repeatedly demonstrated that damage to RBCs caused by storage becomes prominent at the beginning of the 2nd week of storage (Kozlova et al., 2015, 2017; Xu et al., 2018). Furthermore, this damage was noted to progress with increasing storage duration (D’Almeida et al., 2000; Bennett-Guerrero et al., 2007; D’Amici et al., 2007; D’Alessandro et al., 2012, 2015; Gevi et al., 2012; Santacruz-Gomez et al., 2014; Kozlova et al., 2017). As a part of the storage lesion, donated RBCs lose their deformability- reported for the first time (as far as we know) by Kucera et al. (1985). In his study, Kucera et al. (1985) used the filtration technique to characterize the deformability of cells.

**FIGURE 1:**
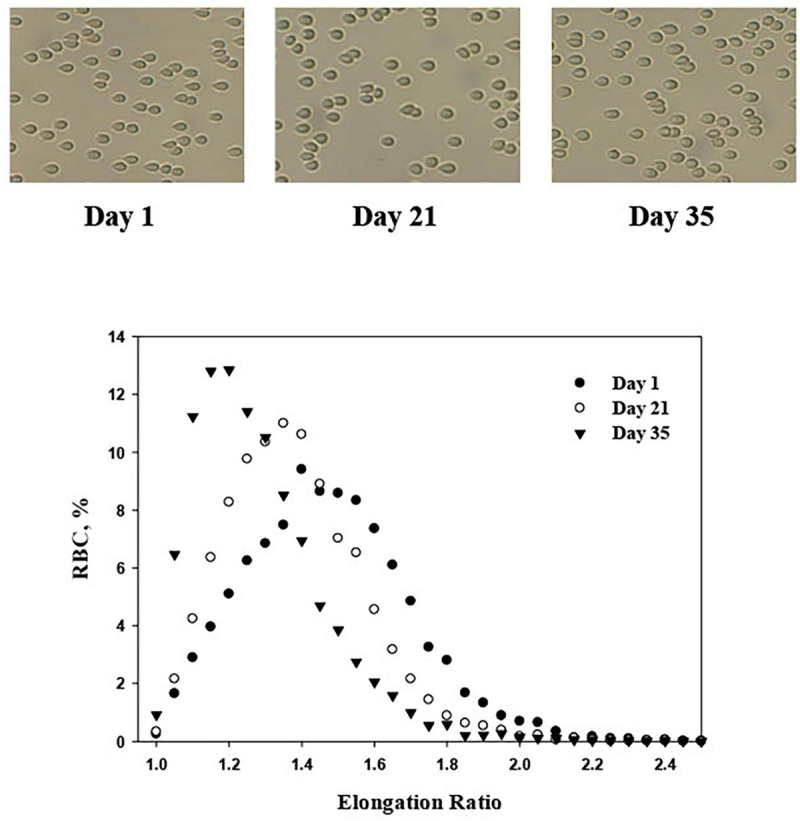
An illustrative micrograph of PRBCs [adhered to glass slide of the flow-chamber (Barshtein et al., 2020b)] under a flow-induced shear stress of 3.0 Pa. Distribution of RBC deformability, expressed by their Elongation Ratio, in RBC population (8’000–10’000 cells), presented by the percentage of RBCs in a discrete elongation ratio value.

Over the years, there have been methods for determining the deformability of cells have become more sophisticated (Mason and Weitz, 1995; Amin et al., 2007; Musielak, 2009; Zheng et al., 2013; Tomaiuolo, 2014; Huisjes et al., 2018). The available experimental methods can be divided into two categories: instruments that measure RBC suspensions (filtration, viscosimetry, and ektacytometry) and single-cell techniques (optical tweezers, microrheology, or micropipette aspiration). Both of these classes have advantages and disadvantages. When measurements are carried out for tens of cells, this makes it possible to characterize the mechanical characteristics of individual cells. However, on the other hand, it does not allow obtaining statistically reliable results due to the significant variability of characteristics. At the same time, both filtering and ektacytometry allow getting averaged features and do not say anything about the distribution of deformability in the RBC population. Characterization of RBCs deformability in flow-systems (flow-chamber) is an alternative method that allows both to assess the features of an individual cell and determine the distribution of indexes in a substantial population (Figure 1). Using of flow-chamber/microfluidic approach allows direct visualization of deformed cells and the distribution of RBC’ deformability in a population from hundreds (Kwan et al., 2013; Cluitmans et al., 2014) to thousands (Relevy et al., 2008; Zheng et al., 2014; Barshtein et al., 2020a, b) of cells.

In the initial period of studying the effect of cold-storage on the deformability of PRBCs was carried out using the filtration method (Kucera et al., 1985; Wegner and Kucera, 1989) and ektacytometry (Cluitmans et al., 2012; Sosa et al., 2014; Marin et al., 2021; van Cromvoirt et al., 2021). In the last decade, measurements with a flow chamber (Relevy et al., 2008; Barshtein et al., 2014, 2020b; Orbach et al., 2017) or microfluidic systems (Guruprasad et al., 2019; Islamzada et al., 2020; Man et al., 2020) have become more frequently used.

Microrheology has been widely applied for measuring single-cell viscoelasticity. This technique accounts for various passive and active particle-tracking methods developed to measure viscoelasticity at a subcellular level by analyzing particle trajectories. A mean-squared displacement (MSD) is determined from particle trajectories and relates to the creep compliance based on the generalized Stokes-Einstein relation (Mason and Weitz, 1995).

The reader can find a detailed analysis of techniques for characterizing erythrocyte deformability in the following reviews (Musielak, 2009; Zheng et al., 2013; Tomaiuolo, 2014; Huisjes et al., 2018).

### What Do We Know About the Deformability of Stored RBCs?

Over the current decade, the number of publications devoted to studying changes in the deformability of RBCs during storage and identifying the factors affecting this process has significantly increased. Moreover, the problem of storage lesion is simultaneously considered from the purely practical (quality control of packed RBCs units), optimization of the storage protocol, and so on) and the scientific (identification of mechanisms behind this phenomenon, etc.) points of view.

Table 1 presents the results of several selected studies in which changes in the deformability of packed red blood cells (PRBCs) during storage are discussed. Despite the wide variety of methods used by different authors for characterizing PRBC deformability, the predominant conclusion is to confirm the results established by Kucera et al. (1985). The general implication from most of the cited publications is that cell deformability begins to decrease after 14–21 days of storage. However, the significance of this effect strongly varies.

**TABLE 1:** Deformability of packed RBC during their storage.

**Storage medium**	**Leukofiltration YES/NO**	**Irradiation**	**Number of tested units**	**Conclusion**	**References**
MAP	No	X-ray	5	The deformability of PRBC was reduced during storage, and the decrease was further enhanced by x-ray irradiation. In addition, irradiation of the PRBCs unit leads to an increase in the proportion of non-deformed cells	Suzuki et al., 1996
MAP	No	Gamma	5	The decreased deformability of γ-ray-irradiated PRBCs during storage was predominantly caused by cell dehydration	Cicha et al., 2000
SAGM	No	No	6	The deformability of PRBCs is not affected during storage in the blood bank for up to 5 wks	Raat et al., 2005
SAGM or PAGGSM	Yes	No	20	Decreased RBC deformability was similar for both additive solutions	Zehnder et al., 2008
CPDA-1	No	No & Yes	6	Deformability PRBCs are sensitive to cold storage and irradiation, which accelerates cell damage	Relevy et al., 2008
SAGM	Yes	No	10	The deformability of the PRBC did not change during storage	Henkelman et al., 2010
CPDA-1	Yes	No	40	The concentration of ATP is not a valid marker for red cell deformability and may not reflect the *in vivo* survival of TRBCs	Karger et al., 2012
AS-5	No	No	24	RBCs suspensions showed a progressive decrease in erythrocyte deformability. There were no statistically significant differences in erythrocyte deformability between cells from male and female donors	Daly et al., 2014
SAGM	Yes	No	5	The deformability of PRBCs is not affected during storage in the blood bank	Cluitmans et al., 2014
SAGM	No	No	5	The deformation index of RBCs under folding does not change significantly over blood storage	Zheng et al., 2014
SAGM	No	No	8	The deformability of PRBCs demonstrated statistically significant variability among donors and storage capacity	Matthews et al., 2015
AS1 or AS3	Yes	No	34	The deformability of PRBC decreased with storage duration and was not correlated with oxidative stress markers	Nagababu et al., 2016
SAGM	No	No	7	PRBCs stored up to 21 days were able to restore their deformability and ATP level to values close to those of donated RBCs, whereas older RBCs (28–42 days), even after recovery, had significantly low deformability and ATP level than donated RBCs	Xu et al., 2018
SAGM	No	No	3	The most substantial decrease in PRBC deformability occurs during the 4th week of storage. The authors discuss the relationship between morphological changes in PRBCs and their deformability	Geekiyanage et al., 2020
SAGM	NO	No	12	The deformability of PRBCs showed a significant alteration with storage while donor gender did not reach a significant effect	Mykhailova et al., 2020
SAGM	Yes	No	20	The deformability of freshly collected RBCs exhibited marked variability already on the day of donation. In addition, the aging curve of packed RBC deformability varies significantly among donors	Barshtein et al., 2020b
SAGM	Yes	No	8	The aging curve of RBC deformability varies significantly across donors but is consistent for each donor over multiple donations	Islamzada et al., 2020
AS-3	Yes	No	9	Small subpopulations of poorly deformed red blood cells significantly reduce the ability of entire populations of PRBCs to cross microfluidic networks and significantly increase the frequency and duration of red cell blockage events	Piety et al., 2021

Raat et al. (2005) explained this discrepancy by the inherent difficulty in comparing results obtained by different experimental conditions and the lack of an appropriate calibration standard. As follows from the data presented in Table 1, the authors of publications have used different storage mediums and methods of PRBCs unit preparation.

Moreover, as follows from the data in Table 1, there is a significant variation in the sample sizes of the tested blood units between publications. The statistical reliability of the results obtained determines the adequacy of the conclusions reached by the authors.

Several publications, which report no change in the RBC deformability during cold storage, clearly stand apart from the rest (Raat et al., 2005; Cluitmans et al., 2012; Zheng et al., 2014; Barshtein et al., 2020b; Islamzada et al., 2020). The authors of these publications did not record any deterioration in cell deformability for the entire group (Raat et al., 2005; Cluitmans et al., 2012; Zheng et al., 2014) of the study samples or some of them (Barshtein et al., 2020b; Islamzada et al., 2020).

Thus, taking into account the relevant reservations, and based on the results obtained by various research groups, the following general conclusions can be formulated:

• In an overwhelming number of published studies, regardless of the measurement method, RBC storage leads to a decrease in their deformability. However, the ability of PRBCs to maintain their deformability during storage varies significantly between donors.
• A noticeable deterioration in PRBCs deformability begins after the end of the 2nd week of storage.
• The slightest damage to the deformability of RBCs during storage is caused when leukofiltration is implemented during the preparation of the unit, and the storage is carried out in SAGM (saline–adenine–glucose–mannitol) solution.
• Deformability of RBC at all stages of processing/storage in the blood-banking practice possesses significant donor-to-donor variation.
• The gender and the donor’s age have a significant impact on the ability of RBCs to maintain their deformability during storage.
• The ability of RBCs to retain their deformability during storage depends mainly on the anti-oxidative status of the donated blood unit.
• Treatment of RBCs with a rejuvenating solution (e.g., Rejevisol) or with the human serum allows a partial restoration of cell deformability’s initial (pre-storage) level.

In the following sections of the review, we will consider some specific aspects associated with the deformability of PRBCs.

### Unit-to-Unit Variability of RBC Deformability

In recent years, the literature has widely discussed the unit-to-unit variability of PRBCs features (Tarasev et al., 2014, 2015; Sparrow, 2017; Barshtein et al., 2020b; Melzak et al., 2021). PRBCs from different units differ significantly depending on the donor, the condition of the collection and processing of blood, and the PRBC unit storage conditions. Despite ongoing efforts to minimize variations in the processing of units (Garraud and Tissot, 2018), the significant disparity is still present.

Recently, much attention has been invested in identifying the effect of donor-related factors on the functionality of collected PRBCs and the stability of cells during cold storage (Daly et al., 2014; Hazegh et al., 2020; Mykhailova et al., 2020; Nemkov et al., 2020; Tzounakas et al., 2020). In these studies, it was found that the age, gender, body weight (as well as other characteristics) of the donor could significantly impact the functionality of donated RBCs and their stability during storage. The identified relationship between age, sex, body weight, and additional donor parameters, on the one hand, and the properties of donated RBCs, on the other hand, lead to significant variability in PRBCs functionality (Dern et al., 1966; Tarasev et al., 2014, 2015; Tzounakas et al., 2016; D’Alessandro et al., 2019; van Cromvoirt et al., 2021). One possible explanation of this phenomenon is that the properties of each blood unit are affected by both genetic (D’Alessandro et al., 2015, 2017; Nemkov et al., 2016) and non-genetic (Tzounakas et al., 2016) donor-related factors (Garraud and Tissot, 2018). Garraud and Tissot (2018) have described additional donor-to-donor variations: the time of donation (morning and afternoon), after a meal or following fasting, drug, and dietary supplement intake, menstrual cycle (for non-menopausal women), diet, and many others (Tzounakas et al., 2016).

Research directly targeting unit-to-unit variations in PRBCs deformability has only recently been initiated, with several authors have demonstrated the significance of this phenomenon (Frank et al., 2013; Matthews et al., 2015; Guruprasad et al., 2019; Jeon et al., 2019; Barshtein et al., 2020b; Islamzada et al., 2020; van Cromvoirt et al., 2021). It was found that two factors primarily cause variability among units: the difference in the initial properties of donated RBCs and the differing ability of the cells to retain their deformability during storage (Barshtein et al., 2020b; Islamzada et al., 2020).

There are two types of “aging curves” describing the kinetics of change in the cell deformability during storage (Barshtein et al., 2020b; Islamzada et al., 2020). So, Barshtein et al. (2020b), by visualization of cells in the flow-chamber, demonstrated that in one case, the percentage of low-deformable cells (% LDFC) significantly changes after 2 weeks of storage, while the other with the % LDFC remains stable throughout the entire storage duration. The ratio between the number of blood units belongs to the first or the second type was 1:1 (Barshtein et al., 2020b). Similar results were obtained by Islamzada et al. (2020) using cell sorting.

#### Interim Conclusions

So far, from the results presented above, we can conclude that donated blood is characterized by high donor-to-donor variability in RBC deformability and that this variability applies to the PRBC units as well. It follows that for analysis of PRBCs stability during storage, it is more justified to consider not the average (over the entire pool of units) but an individual (per-unit) “aging-curves.” Thus, the patterns described above support those authors who considered the reliability of storage duration (age) as a sole factor defining a unit quality (Koch et al., 2019; Yedgar et al., 2019). With that in mind, there is a reason to hope that the joint efforts of various research groups will lead to developing a clinically applicable model of the bag’ inventorying in a blood bank, based on scientific criteria instead of the FIFO principle universally utilized today.

### Reversibility of Storage-Related Impairment in RBC Deformability

When a recipient is transfused with PRBC, erythrocytes with a specific set of properties are injected into his bloodstream. Heavily damaged senescent cells are removed from the bloodstream shortly after transfusion (Luten et al., 2008; Bosman, 2013; Nagababu et al., 2016; Barshtein et al., 2017). This type of cell includes low-deformable RBCs (Barshtein et al., 2017).

However, most transfused red blood cells (TRBCs) remain in the recipient’s circulation (more than 75% according to Bosman (2013) and Roussel et al. (2018) in a state that differs significantly from storage conditions and partially recovers their properties (Barshtein et al., 2018a). This allows them to stay in the recipient’s blood for an extended period. Since the duration of the post-transfusion restoration can be regulated by several physiological factors, the period needed for full recovery varies widely from several hours to several days (Valtis, 1954; Beutler and Wood, 1969; Valeri and Hirsch, 1969; Enoki et al., 1986; Heaton et al., 1989). However, long recovery times (tens of minutes) increase the likelihood that the spleen will clear TRBCs from circulation.

Ichikawa et al. (2019, p. 125) compared the deformability of patients’ RBCs before and after cardiac surgery with cardiopulmonary bypass. They demonstrated that RBCs deformability decreased during surgery, but the most significant elevation in cell rigidity occurred after PRBC transfusion. Frank et al. (2013) compared the deformability of the recipient’s cells before and after PRBCs transfusion. The authors (Frank et al., 2013) demonstrated that the deformability of the patients’ red cells has decreased during transfusion (compared to pre-transfusion state) and that this abnormality was not reversed and got worse (in the case of moderate transfusion volume) over the subsequent 3 days. These data indicate there’s no restoration of the deformability of transfused RBCs *in vivo*. However, under conditions described by the authors, several processes co-occur, such as removing erythropoiesis, restoring TRBC, and, possibly, bleeding, and the balance between these processes will determine the observed result. Due to the complexity of the simultaneous processes taking place after the transfusion of PRBCs *in vivo*, several authors examined the recovery of cell deformability under *in vitro* conditions.

#### Key Factors Capable of Improving the Cell Deformability

One of the critical reasons for improving PRBCs deformability may be the restoration of the intracellular level of ATP, which is significantly reduced during cold storage (Hess, 2014). And although there are conflicting data (Weed et al., 1969; Karger et al., 2012; Huisjes et al., 2018) on the relationship between intracellular ATP content and RBC deformability, some authors have tested the effectiveness of restoring the expected level of ATP in reducing PRBC stiffness. Thus, Xu et al. (2019) examined whether stiffness of PRBCs can be restored in human serum (at temperatures below 37°C) and the role of storage duration in this process. Authors demonstrated that PRBCs could recover their deformability and ATP concentration in human serum, with the extent of recovery decreasing as a function of storage duration (Xu et al., 2019). Moreover, the recovery processes took place on a scale of tens (10 – 90 min) of minutes, and in the case of a more extended storage period, a longer recovery was required.

Unlike, Barshtein et al. (2018a) and Xu et al. (2019) carried out experiments in the Rejuvesol medium. This medium is a sterile, non-pyrogenic aqueous solution of sodium pyruvate, inosine, adenine, dibasic sodium phosphate, and monobasic sodium phosphate being used for extracorporeal rejuvenation of PRBC units. Rejuvenation is accomplished by incubating the contents of one 50 mL vial of Rejuvesol with one unit of PRBC for 60 min at 37°C. PRBC rejuvenated before 6 days of storage may achieve ATP levels over 1.5 times normal. Thus, Meyer et al. (2011) conclude that Rejuvesol can restore ATP levels in PRBCs stored up to 120 days in AS-1 or AS-3.

Barshtein et al. (2014, 2018a) demonstrated that Rejuvesol treatment causes partial repair of PRBC deformability and the degree of reversal is inversely proportional to the extent of damage, where some of the changes could no longer be reversed. In contrast, D’Almeida et al. (2000) observed a complete (up to the control levels) restoration of RBC deformability following treatment of PRBCs with Rejuvesol.

#### Interim Conclusions

Thus, we can summarize that due to an increase in the intracellular level of ATP, there is a complete or partial restoration of PRBCs original deformability. The degree of recovery is inversely proportional to the severity of damage sustained by the erythrocytes during storage. The development of irreversible changes in the deformability of PRBCs may be mainly associated with the process of vesiculation (see below), which leads to permanent alteration in the membrane composition/structure and the shape of the cell.

## Causal Relationship Between the Red Cell Lesion and the Decrease in Its Deformability

The cellular morphology, in addition to the surface/volume ratio, membrane elasticity, and cytoplasmic viscosity, must be considered the main factors affecting the red cell’s response to deforming forces (Huisjes et al., 2018). Most of these characteristics change at one stage or another during the cold storage of PRBCs, increasing cell stiffness. In this section, we will not go into a detailed discussion of specific mechanisms. Still, we will rather attempt to outline a general cause-consequence relationship between the biochemical/morphological properties of PRBCs (that change during storage) and their deformability.

### The Alteration of RBC Membrane Composition/Structure During Cold Storage

Among several hypotheses, the oxidative stress/free-radical theory offers the best mechanistic explanation of *in vitro* aging of erythrocytes (Pandey and Rizvi, 2010). Erythrocyte aging is accompanied by the inactivation of cellular enzymes (including the anti-oxidative defense enzymes) and many membrane transporters (Bartosz, 1996). Storage conditions lead to (1) cell shape changes from discocytes to spherocytes, (2) increased density, (3) decreased volume, and (4) an increased membrane fragility (Antosik et al., 2015). These alterations are induced by conformational changes of cell proteins (such as band-3, hemoglobin, and spectrin) and by the reduction in lipid fluidity, which influences the cortex viscoelasticity and the bilayer structure.

**FIGURE 2:**
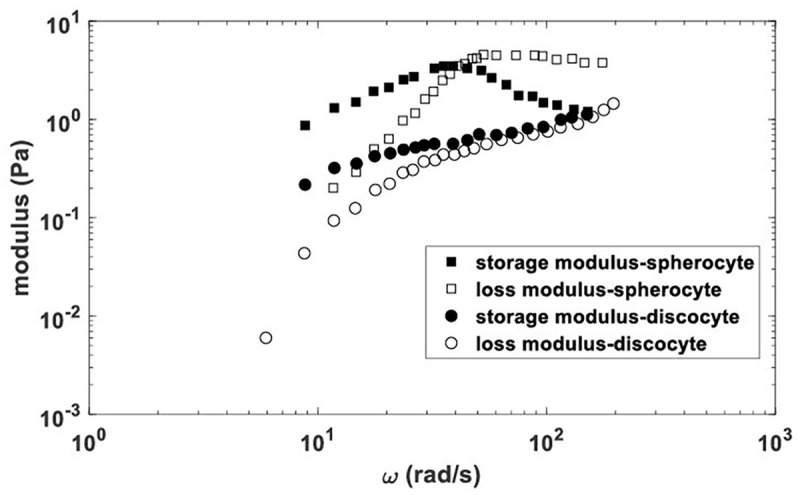
Storage and loss moduli vs. angular velocity for spherocytes and discocytes extracted from microrheological data published by Park (2007) and Amin et al. (2007).

Comparative analysis of the membrane viscoelasticity of (1) young RBC (discocytes) and (2) aged RBC (spherocytes) under the same experimental conditions is shown in Figure 2. This figure shows the frequency sweep data in accumulation and loss moduli versus angular velocity extracted from microrheological measurements (Amin et al., 2007; Park, 2007). Storage modulus quantifies the storage energy, while the loss modulus quantifies the energy dissipation caused by the cell membrane structural changes under thermal fluctuations.

**FIGURE 3:**
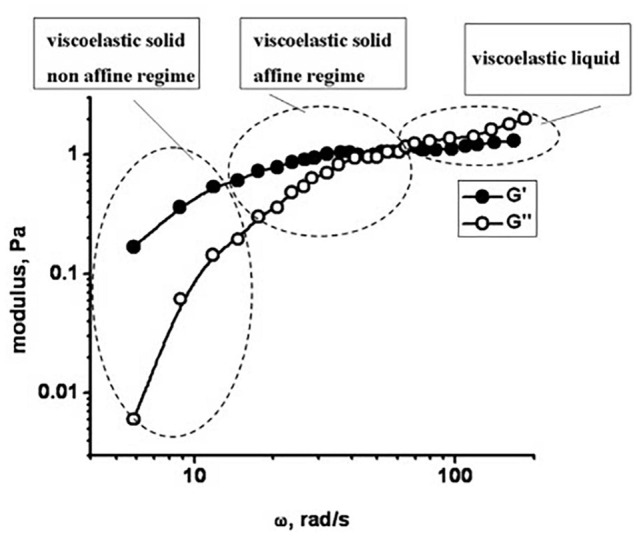
Viscoelasticity of RBC obtained by the microrheological measurements: Storage and loss moduli (G’ and G”) vs. angular velocity.

As follows from the given figure, spherocytes are characterized by higher values of the storage modulus in comparison with discocytes at the same angular velocities, which indicates a greater stiffens of their membrane. In addition, for spherocytes, higher values of the loss modulus (vs. the angular velocity) were obtained and a decrease in the storage modulus (at higher angular velocities), which indicates their more fragile membrane compared to discocytes. Rheological response of the RBC membrane under microrheological experiments can be considered within three viscoelastic regimes depending on angular velocity (i.e., corresponding time scale), as was shown schematically in Figure 3.

The reduction in lipid fluidity, in its turn, is caused by (1) lipid peroxidation (Lukyanenko et al., 2004) and (2) phosphatidylserine (PS) externalization (Koshkaryev et al., 2020). Koshkaryev et al. (2020) have outlined the inter-relations between PS externalization and the rearrangement of band-3 molecules. The rearrangement of band-3 molecules influences cortex viscoelasticity by reducing spectrin filaments’ flexibility (Pajic-Lijakovic, 2015). This flexibility depends on the number of attached band-3 molecules per single filament. Band-3 molecules form two types of complexes: those in the form of tetramers creating higher affinity complexes with ankyrin (dissociation constant of ∼5 nM) (Tomishige et al., 1998; Kodippili et al., 2012) and located near the center of the spectrin tetramers, and those in the form of dimers, creating lower affinity complexes with adducin (dissociation constant of ∼100 nM) (Franco and Low, 2010; Kodippili et al., 2012). The rest of band-3 molecules (∼30%) exist as monomers that are freely mobile under the isotonic conditions, as is the case of young erythrocytes (Tomishige et al., 1998). This mobile fraction of band-3 is capable of forming low-affinity complexes with spectrin.

Oxidative stress induces an increase in the volume fraction of free band-3 molecules and their clustering during aging (Arese et al., 2005). All types of band-3 subpopulations (through these complexes) contribute to the spectrin conformational changes by reducing its mobility, leading to the cortex stiffening (Pajic-Lijakovic, 2015). Hemoglobin (Hb) molecules’ binding to band-3 also induces cortex stiffening, prominent in aged erythrocytes (Kang et al., 2008). This phenomenon is induced by changing the conformational and oxidative states of Hb molecules and their clustering. The average initial Hb concentration for a single intact erythrocyte is 7.3 mol/m^3^ (Delano, 1995). The Hb concentration and the viscosity of its solution increase with a decrease in the cell volume induced by aging. The cell volume decreases by ∼15% for aged erythrocytes as a result of (1) changes in the membrane viscoelasticity and (2) reduction in the K^+^ outflow caused by inactivation of membrane transporters (Nash and Meiselman, 1983). The K^+^ outflow reduction leads to the loss of osmotic water and, as a result, to dehydration of aged erythrocytes. The membrane structural changes result in a decrease in zeta-potential from about −14 mV for young cells to about −10 mV in the aged ones (Chen et al., 2007), with a feedback impact on the distribution of band-3 and Hb molecules.

The spectrin inter-and intra-filament interactions, band-3 rearrangements, and lipid lateral movement under thermal fluctuations influenced by the activity of ATP activity induce an anomalous nature of energy dissipation quantified by fractional derivatives (Pajic-Lijakovic, 2015). Betz et al. (2009) reported that the membrane fluctuates as in thermodynamic equilibrium (i.e., thermal fluctuations) at short timescales (lower than 100 ms). At longer timescales, the equilibrium breaks down, and fluctuation amplitudes are higher by 40% than the membrane equilibrium theory predicted. This increase of the amplitudes was related to ATP activity. Sleep et al. (1999), Yoon et al. (2008), Yoon et al. (2011), and Betz et al. (2009) reported that changes in the non-linear cortex stiffening represent the consequence of breaking of a fraction of actin-spectrin junctions during the deformation process driven by ATP activity. ATP depleted cells (echinocytes) are stiffer than discocytes for the same angular velocities (Pajic-Lijakovic and Milivojevic, 2014). Pajic-Lijakovic and Milivojevic (Pajic-Lijakovic and Milivojevic, 2014) developed a constitutive model that accounts for: the cortex viscoelasticity, bilayer viscoelasticity, and mechanical coupling by considering the timescale from milliseconds to seconds. They accounted for thermal fluctuations dominant at a short timescale and ATP activity dominant at a long timescale (Pajic-Lijakovic and Milivojevic, 2014). As defined by its shear modulus, the age-induced cortex stiffening is quantified by values one order of magnitude higher than those for young cells. Consequently, intensive energy dissipation caused by the cortex and the bilayer rearrangements in the young cells is also quantified by values one order of magnitude higher than those measured in the aged cells. The damping effects caused by the membrane structural changes under thermal fluctuations, which influence viscoelasticity, also increase cell aging (Pajic-Lijakovic and Milivojevic, 2014).

#### Interim Conclusions

In this section, we have demonstrated that a change in the conformational state of band-3 molecules, their clustering, and space distribution (Antonelou et al., 2010; Bosman et al., 2010) can be considered one of the main factors which influence the viscoelasticity of the cortex and the lipid bilayer, leading to a decrease in cell deformability that occurs during RBC storage. We suggest that the reduction in deformability may be due to the altered coupling between the actin-spectrin cortex and the bilayer, which induces a change in membrane organization with an increased PS exposure. Consequently, (1) the distribution of band-3 molecules, (2) viscoelasticity of the cortex, and (3) viscoelasticity of the bilayer altered by ATP activity cause an increase in cell stiffness. Thus, even though we have a general understanding of the relationship between the rearrangement of the membrane (induced by cold storage) and the cell deformability, we still cannot propose a relevant, complex causal model. Therefore, one of the obvious goals of future experimental and numerical studies is to create such a model.

#### Vesiculation of RBC Membranes During Cold Storage

Microvesicles generation is an integral part of the lesion of RBCs during cold storage. These particles, 100 microns in size, contain lipid raft proteins and oxidized or reactive signaling components associated with senescent erythrocytes (Kriebardis et al., 2008). Thus, vesiculation can contribute to removing damaged areas of the membrane, which would otherwise lead to accelerated phagocytosis of RBCs (Bosman et al., 2008; Bosman, 2013).

Although the triggers and mechanisms of microvesicles formation are mainly unknown, there is already a definite view of the forces driving this process. Thus, Freitas Leal et al. (2020) suggests that changes in the organization of membranes, caused by the altered conformation of the membrane protein, constitute the primary mechanism of vesiculation, and precede changes in the organization of lipids. Moreover, Salzer et al. (2008) demonstrated the role of a stomatin-specific, raft-based process in storage-associated vesiculation. The same authors propose a model of RBC vesiculation under cold storage conditions, which considers the raft-stabilizing properties of stomatin and the previously reported storage-related changes in the cytoskeletal organization. At the same time, it has been noted that the low temperature of cell storage stimulates the aggregation of rafts. Microvesicle counts in the RBCs bag are sensitive to the donor characteristics, unit-processing methods, and storage duration (Almizraq et al., 2018; Gamonet et al., 2020).

In this way, microvesicles generation may affect RBC deformability via a dual mechanism: First, it is associated with RBC cytoskeletal reorganization and loss of membrane lipids and proteins such as stomatin and flotillin, which influence the membrane viscoelasticity (Salzer et al., 2008; Orbach et al., 2017). Second, the generation of EVs, which are high in surface but low in volume, can be expected to reduce RBC area-to-volume ratio, which in turn would lead to an increase in the residual stress accumulation within the membrane and to a decreased cell deformability (Salzer and Prohaska, 2001; Baskurt and Meiselman, 2003; Ciana et al., 2017).

Regardless of the possible mechanism of the cause-and-effect interaction, the authors of three experimental studies demonstrated a relationship between the concentration of vesicles in a unit of PRBC and red cell deformability (Acker et al., 2018; Almizraq et al., 2018; McVey et al., 2020). It is important to emphasize that in the two cited publications, the studied blood units were prepared using non-identical protocols, and different techniques were implemented to characterize the deformation of erythrocytes.

#### Interim Conclusions

The above research results indicate that during cold storage of PRBCs, there is an intensive formation of microvesicles (accumulated in the medium) of various sizes and compositions. This phenomenon, leading, in particular, to a decrease in the cell surface and a change in the membrane viscoelasticity, causes an elevation of PRBC rigidity.

## Deformability of Cells as a Quality Marker of PRBCs

As noted above, the functionality of PRBCs from different units varies greatly (Dern et al., 1966; Tarasev et al., 2014, 2015; Tzounakas et al., 2016; D’Alessandro et al., 2019), leading to the significant difference in the effectiveness of transfusion. Moreover, not all PRBC units provide identical post-transfusion benefits (Barshtein et al., 2016, 2017; DeSimone et al., 2020). In particular, transfused RBCs from some units can function for long periods, while others are rapidly eliminated from the recipient’s bloodstream (Bosman, 2013; Dinkla et al., 2014; Tzounakas et al., 2016; Roussel et al., 2018). This situation is highly undesirable for chronic transfusion recipients because of potential adverse effects, such as iron overload (Lal, 2020). Therefore, there is a need to develop reliable biomarkers to attest to the quality of PRBCs and to optimize the management of units stored in the blood bank. The presence of such biomarker (s) will make it possible to identify units containing RBCs capable of adequate long-term functioning in the recipient’s bloodstream (Yedgar et al., 2019). Furthermore, with the help of such biomarker (s), it will be possible to identify those donors who can consistently provide high-quality units and thus make significant progress toward a transition to personal transfusion medicine (Islamzada et al., 2020). A unit of PRBCs with higher functionality can be highly beneficial for patients requiring repeated, life-long transfusions (such as those suffering from thalassemia or sickle cell anemia), in whom the reduction in transfusion requirements (Barshtein et al., 2017) would diminish the side effects associated with continuous blood-products therapy.

For a long time, the storage duration of a blood unit was regarded as the only criterion for PRBCs quality. However, many researchers have questioned this statement (Matthews et al., 2015; Sparrow, 2017; Roussel et al., 2018; Koch et al., 2019; Yedgar et al., 2019). For example, Koch and colleagues (Koch et al., 2019) conclude in their review that the storage duration (“calendar age”) is not an appropriate measure of PRBCs quality and that “a functional measure of stored RBC quality « real age » may be better than the calendar age.” In concordance with this opinion, Yedgar et al. (2019) suggested that the hemodynamic functionality of erythrocytes, particularly their deformability, can be used as a marker of PRBC quality and as a critical indicator of RBC quality and post-transfusion viability. In several additional publications, authors reach the same conclusion (Cluitmans et al., 2014; Barshtein et al., 2016, 2017; Roussel et al., 2018; Geekiyanage et al., 2020; Islamzada et al., 2020). Thus, by *in vitro* experiments, Piety et al. (2021) demonstrated that small subpopulations of low-deformable stored cells significantly decrease the ability of PRBCs to traverse microfluidic networks and increase the occurrence of plugging.

The most prevalent hypotheses for red cell clearance mechanism(s) are the externalization of phosphatidylserine, expression of neoantigens on RBC surface, and elevation of cells rigidity (Thiagarajan et al., 2021). Red cells with greater than normal stiffness have an increased chance of being eliminated by the spleen (Cranston et al., 1984; Duez et al., 2015; Klei et al., 2017; Safeukui et al., 2018), the main red cells quality-controlling organ (Mebius and Kraal, 2005; Deplaine et al., 2011). In addition, the stiffness of RBC has been shown to serve as an indicator for macrophages to initiate phagocytosis leading to RBC clearance (Fens et al., 2010; Sosale et al., 2015).

The existence of a relationship between the deformability of cells and their ability to pass through the spleen has been shown using both numerical modelings (Li et al., 2018) and *in vitro* (Deplaine et al., 2011) and *ex vivo* experiments (Safeukui et al., 2018). However, since an elevation in RBC rigidity is not the only reason that makes it difficult for them to pass through thin slits (Thiagarajan et al., 2021), the authors of several numerical (Pivkin et al., 2016; Li et al., 2018) and *in vitro* (Deplaine et al., 2011) studies discussed the specific weight of this factor. Thus, if not an ultimatum criterion for cell survival, the deformability of cells is undoubtedly an essential factor determining their clearance from the bloodstream.

Moreover, several reports of clinical studies suggest that RBC deformability may serve as a potential biomarker of various types of red cell pathology (Saha et al., 2018; Farber et al., 2020; Lu et al., 2020; Tan et al., 2020; Porro et al., 2021). However, as far as we know, testing this hypothesis in a clinical setting has only recently begun (Barshtein et al., 2016, 2017).

For example, Barshtein et al. (2016, 2017) have examined the correlation between deformability of transfused PRBCs and transfusion-related outcome for β-thalassemia major patients. In this study, to characterize PRBC deformability, a cell flow analyzer was used to measure the elongation index (under a shear stress of 3.0 Pa) of individual cells and the distribution of this parameter in the population of 6000–8000 cells. The authors demonstrated that deformability of transfused PRBCs define transfusion-related outcome (Barshtein et al., 2016, 2017). Thus, the proportion of low-deformable cells was inversely correlated to the transfusion-induced increment in hemoglobin value (Barshtein et al., 2017) and improved recipient blood skin flow (Barshtein et al., 2016).

### Interim Conclusions

The above results indicate that cell deformability can be implemented as a potential biomarker when assessing the quality of PRBC units. This will allow the quality of each collected unit of PRBC to be quantified. The introduction of such an approach into the blood banking practice will enable optimizing the process of inventorying units and proceed toward a personal approach when selecting units for each recipient.

## Limitations

The number of publications on this topic is constantly growing, and in the last decade, more than a hundred studies have been published annually (PUBMED). Consequently, the review presented here is not intended to cover the full range of problems associated with PRBC deformation. We have focused only on those aspects that seem to us to be the most important for this issue.

In addition, we deliberately left out of the discussion of methods used by different authors for determining the cell deformation and their influence on the results obtained by them, since this issue has been extensively covered in the literature (Baskurt et al., 2009; Musielak, 2009; Nemeth et al., 2015; Depond et al., 2019).

Still, despite the above limitations, we believe that our review will allow the reader to get acquainted with the main directions of research in the deformation of stored PRBCs and the key factors that may regulate this function. In addition, we attempted to outline some of the possible directions for future research in this area. Moreover, our explicit intention was to draw attention to the significance of the variability of cell deformability both among donors and PRBC bags, stressing the controversy of the first-in-first-out approach universally accepted in blood banking practice for the management of stored PRBCs.

## Futures Perspectives

The question of the effect of storage on cell deformability has a long history. As mentioned above, research in this area began in the mid-1980s. At present, with the advent of new approaches (microfluidics) which enable us to determine the distribution of deformability in the cell population (Relevy et al., 2008; Guruprasad et al., 2019; Barshtein et al., 2020b; Islamzada et al., 2020; Man et al., 2020), the scientific interest in this field has increased. This led to an increase in the number of research groups working in this area.

Most researchers observed a significant deterioration in cell deformability after 2 to 3 weeks of storage (see Table 1). At the same time, for several units, the stability of deformability was observed throughout storage (Barshtein et al., 2020b; Islamzada et al., 2020). There was also significant variability in the deformability of the red cells, both between different donors and between PRBC units. In addition, it was found that the preparation of the PRBC unit causes an alteration in the deformability of cells (Barshtein et al., 2020a).

The deformability of PRBCs is interesting not in themselves but because of their physiological role (Cluitmans et al., 2014, p. 26; Roussel et al., 2018, p. 57; Geekiyanage et al., 2020, p. 54; Islamzada et al., 2020, p. 105; Islamzada et al., 2020, p. 34) and how this feature affects the post-transfusion benefit of the blood unit recipient. Thus, several researchers (Luten et al., 2008; Bosman, 2013; Nagababu et al., 2016; Barshtein et al., 2017) have shown that cell deformability determines the removal rate TRBCs from the recipient’s circulation after transfusion. This concept was later strengthened by a study reported by Barshtein et al. (2016, 2017), showing that RBC deformability and transfusion outcomes in β-thalassemia patients are correlated.

## Author Contributions

IP-L and GB wrote the manuscript. AG edited the manuscript. All authors contributed to the article and approved the submitted version.

## Conflict of Interest

The authors declare that the research was conducted in the absence of any commercial or financial relationships that could be construed as a potential conflict of interest.

## Publisher’s Note

All claims expressed in this article are solely those of the authors and do not necessarily represent those of their affiliated organizations, or those of the publisher, the editors and the reviewers. Any product that may be evaluated in this article, or claim that may be made by its manufacturer, is not guaranteed or endorsed by the publisher.
